# New insights in the management of pseudomyxoma peritonei

**DOI:** 10.1002/jso.27842

**Published:** 2024-08-29

**Authors:** Clément Pastier, I. H. J. T. De Hingh, Diane Goéré

**Affiliations:** ^1^ Department of Digestive and Endocrine Surgery, Saint‐Louis Hospital AP‐HP Université Paris Cité Paris France; ^2^ Department of Surgery Catharina Hospital Eindhoven The Netherlands

**Keywords:** appendix neoplasm, cytoreductive surgery, hyperthermic intraperitoneal chemotherapy, peritoneal carcinomatosis, peritoneal pseudomyxoma

## Abstract

While a rare entity, peritoneal pseudomyxoma treatment evolves. Decision‐making criteria improve with imaging development and exploratory laparoscopy. Surgery remains at the core of the therapeutic strategy whatever disease progression. Complete cytoreduction plus hyperthermic intraperitoneal chemotherapy (HIPEC) is standard of care. Iterative cytoreduction or debulking is sometimes justified. Intraperitoneal chemotherapy modalities change with early postoperative HIPEC or pressurized intraperitoneal aerosol chemotherapy. Systemic or local treatment such as new chemo/immuno‐therapies or BromAc should improve outcomes. Expertise and multicentric cooperation are more than ever needed.

## 1 INTRODUCTION

Peritoneal pseudomyxoma (PMP) is a rare condition affecting about 2 per 1 000 000 persons each year. PMP is defined by the presence of mucinous ascites of variable abundance, associated or not with epithelial cells, with a variable degree of malignancy.^1^, ^2^ PMP is a clinical entity that has multiple different anatomic sites for its origin even if mucinous appendiceal tumor (LAMN) ruptured into the free peritoneum is the main etiology in 90% of cases.^3^, ^4^

The morphologic appearance (grade) and extent (stage) of the disease are prognostic factors in PMP of appendiceal origin and determine the therapeutic strategy. The grade of the primitive tumor and its peritoneal extension are usually similar.^5^ With numerous classifications published in the last 10 years, while the most recent WHO classification (5th edition, 2019) is widely used by pathologists and oncologists, cellularity of mucus should be considered to make a knowledgeable prognostic assessment after surgery.^6^, ^7^

Accumulation of mucin in the peritoneal cavity can lead to massive symptomatic abdominal distension associated with mechanical and functional bowel dysfunction. In this situation, even if complete cytoreductive surgery is not feasible, debulking could improve quality of life and prolong survival.^8^, ^9^

Complete cytoreductive surgery (CRS) followed by hyperthermic intraperitoneal chemotherapy (HIPEC) is currently the standard of care for pseudomyxoma peritonei as recently recommended by a consensus of international experts.^10^ However, because of the extent of the disease, especially recurrent disease for which CRS would be difficult and risky, or for which complete resection is not technically possible, new approaches have been reported in recent years. The aim of this review was to describe each of these approaches in detail.

## 2 PERITONEAL DISEASE AMENABLE TO COMPLETE CYTOREDUCTIVE SURGERY

### 2.1 Complete cytoreductive surgery and HIPEC

Complete cytoreductive surgery (CRS) followed by HIPEC is the standard of care treatment for PMP.^11^, ^12^, ^13^ The combination of CRS followed by HIPEC has improved patient survival compared to surgical treatment alone with iterative debulking, even if there is no prospective randomized controlled trial comparing these two modalities.^11^, ^14^ Currently, median overall survival after CRS and HIPEC ranges from 97 to 111 months, reaching 196 months (16.3 years) in the multicentre series published in 2012 that included 2218 patients.^15^ Recurrence‐free survival is also high, reaching 98 months (8.2 years).

Regarding the place of HIPEC, there is no randomized trial available comparing CRS without HIPEC and CRS plus HIPEC. However, an analysis from the PSOGI of 1924 patients who underwent surgery for PMP of appendiceal origin between February 1993 and January 2018 showed that patients who underwent CRS followed by HIPEC had significantly better survival compared to patients who underwent CRS alone.^14^ In addition, the addition of HIPEC to CRS was not associated with an increased risk of severe complications, re‐interventions, or mortality at Day 30 and Day 90. Nonetheless, this retrospective study presented limits such as the long period of inclusion that exposed to heterogeneity quality of surgical procedures and variability of HIPEC protocols.

CRS consists of the following steps. At laparotomy, the diagnosis of PMP is confirmed macroscopically by the typical mucinous appearance of the lesions. Next, the extent of the disease is assessed using Sugarbaker's Peritoneal Carcinomatosis Index (PCI). The surgeon decides whether or not to carry out a complete resection (CC0 or CC1) of all the lesions, while “controlling” the risk of complications and the expected postoperative quality of life. Usually only the affected peritoneum is resected, and a systematic complete peritonectomy of all five sectors (greater omentectomy; left upper quadrant peritonectomy; right upper quadrant peritonectomy; lesser omentectomy; pelvic peritonectomy associated or not with splenectomy and cholecystectomy) is not typically performed, but this notion remains debated.^16^, ^17^, ^18^

Negative prognostic factors are incomplete cytoreductive surgery, advanced PCI, high‐grade PMP, preoperative chemotherapy, presence of signet‐ring cells, age >55 years, high biomarkers levels, and postoperative complications.^19^ Furthermore, expertise with an annual volume of 17 cytoreductive surgical procedures impacts prognosis as published by Kusamura et al.^20^

### 2.2 Laparoscopic CRS and HIPEC

Minimally invasive, laparoscopic, or robotic‐assisted CRS with HIPEC (L‐CRS + HIPEC) is currently not recommended as a standard of care. While numerous retrospective studies are reported for ovarian cancer CRS, few reports argued for its use in PMP.^21^ Furthermore, losing the ability to feel the nodules in the peritoneal cavity increases the risk of leaving peritoneal implants behind which may lead to early relapses and decreased survival.^22^

In terms of feasibility, Esquivel et al. first reported a single‐center experience of 14 patients undergoing L‐CRS + HIPEC for non‐ovarian neoplasia (80% appendiceal neoplasms) in 2011. One patient failed due to extensive carcinomatosis and three patients were converted to laparotomy (one for infeasible omentectomy due to BMI 47, one PCI 13, and one mucinous deposit not resectable by laparoscopy).^23^ In a prospective study, Cho et al. reported a 41% failure rate of L‐CRS + HIPEC mainly due to an underestimation of the PCI.^24^ For feasibility, surgeons agreed to reserve a laparoscopic approach for patients with low PCI < 10 and low‐grade disease.

To perform this complex procedure, operating room logistics need to be improved. Authors have published videos with tips and tricks that often require two screens (one on each side of the patient), 30–45° or flexed cameras, multiple port placement, and position changes with the patient secured on the operating table.^25^, ^26^, ^27^ Regarding multiple port placement, it exposes to numerous local recurrences within port sites that are difficult to treat. There are no data addressing this issue.

In 2021, Arjona‐Sanchez et al. published the PSOGI experience including 143 L‐CRS + HIPEC.^28^ The most common indications were low‐grade appendiceal mucinous neoplasm (LAMN) leading to low‐grade pseudomyxoma peritonei (LG‐PMP) in 55% of cases and multicystic peritoneal mesothelioma (MPM) in 14%, with a median PCI of 3. A median of 1 peritonectomy (with a maximum of 4) over 5 quadrants was performed, along with 34.5% bowel resection and 4.1% splenectomy. Data were encouraging in terms of shorter length of stay (median 6 days), similar 30‐day morbidity (36%) or mortality (0.7%). The median time to return to intended oncological treatment was 4 weeks for aggressive histology. Similar data have been reported by Rodríguez‐Ortiz.^29^ However, an improvement in return to intended oncologic treatment did not translate into better survival rates in other malignancies.^30^

Retrospective studies with propensity score‐matched analysis compared outcomes between open and laparoscopic approach. Bortoli et al. compared 13 laparoscopic to 32 open CRS + HIPEC matched for age, ASA, comorbidities, prior surgical score (PSS), and PCI. No difference in terms of duration, complexity of surgery, severe morbidity, reintervention, or 90 days readmissions were demonstrated. However, blood loss, overall morbidity, and postoperative recovery were statistically improved in the L‐CRS + HIPEC.^31^ In another matched analysis by Wang et al. of 33 cases, PFS was not statistically different between groups.^32^ That echoed other studies.^33^, ^34^, ^35^ Even if the oncological benefits of L‐CRS + HIPEC over open remained unclear, its feasibility and safety seems favorable in expert centers with high selection criteria.

“Risk‐reducing” L‐CRS + HIPEC in high‐risk patient of peritoneal relapse such as T4 LAMN or T4 perforated colon cancer could be the most valuable indication for the minimally invasive approach.^36^ This indication concerned 57 patients excluded from the 2021 PSOGI report.^28^ If confirmed in a future study, risk‐reducing L‐CRS + HIPEC could become the standard of care in this selected situation.

### 2.3 Two‐stage cytoreductive surgery

Recently, a two‐stage CRS (2S‐CRS) has been proposed for low‐grade PMP when the disease is too extensive and the surgery too invasive to be performed in a single procedure, for whom the morbidity and mortality of one‐stage CRS (1S‐CRS) would be high.^37^ In this recent study, inclusion criteria were: PCI greater than 20, low‐grade PMP, easily detachable mucinous implants, involvement of the serosa of the small bowel or colon requiring at least 3 resection‐anastomosis. Eight patients were reported with a median PCI of 25. Residual disease of the remaining implants at the end of the first stage was less than 5 mm in thickness in residual implants. Oxaliplatin‐based HIPEC was performed at the end of the first cytoreduction. The median interval between the two steps was 4 months. All patients completed the two‐stage strategy. All patients had a complete histopathological response in the specimens taken from the residual sites during the second stage of surgery. There was no treatment‐related mortality. There was one Clavien‐Dindo grade 3 event per stage. At a median follow‐up of 29.5 months, all patients were alive and free of recurrence.

Another team also reported encouraging results from 2S‐CRS, but with a different approach.^38^ In this series of eight patients, the median PCI was 33. The first step was a complete CRS of the inframesocolic compartment without HIPEC. Colon resection, major omentectomy, lower abdominal peritonectomy, and removal of any pelvic or retroperitoneal masses were usually performed at this stage. Any supramesocolic dissection was minimized to avoid future adhesions. In most cases, a total colectomy with ileorectal anastomosis below the level of the Douglas pouch was performed. Patients were then allowed to recover until the second stage was performed, with a median delay of 111 days (90–212 days). The second step consisted of a thorough adhesiolysis of the inframesocolic compartment with the removal of any nodules that may have recurred in the interval since this first procedure. The pelvis was dissected to allow adequate intraperitoneal chemotherapy diffusion. Major recurrence and invasive nodules were contraindications for CRS and HIPEC. During the second step, complete CRS of the supramesocolic compartment was performed followed by HIPEC. One patient was considered unresectable at the second surgery. Major morbidity was 0% for the first step and 25% for the second step, with no mortality which is lower than other series of extensive PMP.^39^ Therefore, the authors hypothesized that mortality and major morbidity are not cumulative between the two steps. Median follow‐up was 53.8 months (3–73 months). Two patients showed a peritoneal relapse at 9 and 16 months after the complete planned 2S‐CRS. A third step CRS was achieved in one of the two patients. This study demonstrated that 2S‐CRS management of low‐grade PMP patients with very high PCI was safe and feasible, with acceptable postoperative morbidity and no compromise in oncological outcomes.

This rarely performed and reported approach in 2 steps can achieve CC0 cytoreduction, with oncological safety, and should be part of the therapeutic armamentarium in patients with extensive low‐grade PMP. Quality of life seems to be preserved.^40^ It could be a valuable alternative to palliative debulking. The optimal timing for the second step procedure needs further study.

### 2.4 Neoadjuvant and/or adjuvant systemic chemotherapy

PMP may be a low‐grade appendiceal neoplasm (LAMN) or a mucinous appendiceal adenocarcinoma (MACA). Oncological results of a surgical treatment strategy are high.^41^ Added value of prolonged systemic chemotherapy is therefore questionable along with a certain negative impact on quality of life. Studies argued about pseudomyxoma being resistant to systemic chemotherapy.^39^ No randomized study addresses this topic. For more complexity, RECIST response to chemotherapy is still difficult to evaluate.

For resectable PMP, preoperative systemic chemotherapy is generally not considered to be beneficial. A recent Asian study reported about 228 patients receiving preoperative chemotherapy (more than two cycles) before CRS + HIPEC compared to 522 upfront surgery. Seventy percent of the cohort concerned low‐grade PMP. On multivariate analysis, systemic chemotherapy failed to enhance overall survival whereas PCI, completeness of cytoreductive surgery, HIPEC, or pathological grade did have a positive impact.^42^ A recent American study reported 140 patients diagnosed with high‐grade PMP, with 46% receiving at least two cycles of chemotherapy. Preoperative chemotherapy was not associated with less disease burden, better cytoreduction rates, or improved clinical outcomes, regardless of histopathologic subtype.

In fact, few studies showed the benefit of chemotherapy in borderline resectable cases.^43^ In 2010, Sugarbaker et al. published a prospective study concerning 34 patients with resectable high‐grade PMP treated with oxaliplatine plus 5 FU. Preoperative chemotherapy, which caused a complete or near complete response in 10 patients, permitted a significant reduction in PCI and in the complexity of the surgical procedure (such as peritonectomies) and no increase in postoperative morbidity and mortality. Survival was significantly improved in patients who responded (*p* = 0.032).^44^ Further studies with a comparative arm in a homogeneous selected population are needed to make conclusions about preoperative chemotherapy.

Limited data are published concerning postoperative chemotherapy. Strach et al. reported the benefit of chemotherapy in a highly selected N+ patient for overall survival.^45^ A current phase II study is evaluating the benefit of adjuvant capecitabine in the subpopulation of *KRAS* mutated patients with PMP treated by CRS + HIPEC (NCT05321329). KRAS or GNAS mutations were associated with worse progression‐free survival in previous analysis.^46^

A better understanding of prognostic factors would make it possible to target populations for adjuvant treatment. Modern chemotherapy, biotherapy and immunotherapy are constantly evolving, and this raises the question of the potential value of systemic treatment before, during, or after CRS. Given the rarity of PMP, this will require an international collaborative effort of expert centers

## 3 PERITONEAL DISEASE NOT ACCESSIBLE TO COMPLETE CYTOREDUCTIVE SURGERY

In expert centers, 29% of all PMP remain unresectable despite advanced multidisciplinary approaches. This percentage is irrespective of the grade.^47^ For PMP cases that are not accessible to CRS, different treatment options may be proposed, ranging from systemic or intraperitoneal chemotherapy to debulking surgery.

### 3.1 Debulking surgery

When complete cytoreductive surgery is not feasible, either because of patient‐related contraindications or because of the extent of peritoneal disease inaccessible for complete resection, debulking surgery performed in expert centers may help to improve disease‐related symptoms. It aims to resect most of the disease and/or masses responsible for painful or compressive symptoms and improve the patient's quality of life, while being conservative enough to limit postoperative mortality and morbidity.^8^, ^9^

In 2004, Glehen et al. first published a 30‐year experience of 174 patients with incomplete cytoreduction from 645 procedures for PMP. Median survival reached 20.5 months.^48^ Interestingly, repeating procedures improved oncological outcomes. Nonetheless, advanced high prior surgical score (PSS) is associated with less favorable outcomes, raising the question of the risk‐benefit ratio.

In the series from Dayal et al. published in 2013, a maximal tumor debulking was performed in 205 patients, if complete CRS could not be achieved. Thirty percent presented a high‐grade disease with high rates of preoperative biomarkers. Debulking surgery was associated with mitomycin C HIPEC in 62.9%. A majority of patients underwent a greater omentectomy with intestinal resection. No difference existed for any type of postoperative complications. Median overall survival was 32.8 months while it was not reached in the complete CRS group. Significant difference existed between high‐ and low‐grade histology for survival.

In the series from Delhorme et al., the policy was to maximize tumor debulking, leaving less than 20% of the disease in areas where it is unlikely to cause symptoms.^9^ Median PCI was 32/39 and complete CRS was considered unachievable mainly because of diffuse peritoneal involvement requiring multiple organ resection. Median duration of surgery was 330 min and resection of more than 80% of the disease burden was achieved in 23 patients (59%). Median time to symptom recurrence was 23.2 months after debulking. At a median follow‐up of 24.5 months after debulking, half of the patients (*n* = 18) were free of PMP‐related symptoms. One patient reported a chronic digestive fistula.

In a recent meta‐analysis of 766 debulking procedures published in 2020, Zhou et al. reported an overall survival of 40% at 5 years. The prognosis of patients with low‐grade pathology was better than that of patients with high‐grade pathology (HR = 0.54, 95% CI [0.33, 0.89], *p* = 0.01), which is consistent with data from the literature.^8^, ^9^

Given the outcome of these studies, debulking surgery appears to be beneficial in highly selected patients for controlling symptoms and time off treatment, especially for low‐grade PMP.

### 3.2 Debulking surgery followed by HIPEC

As described in the series published by Dayal et al., HIPEC can be added to debulking surgery for PMP noneligible for CRS.^8^ Few studies reported the added value of HIPEC in debulking in PMP while the concept is reported in ovarian cancer.^49^

In 2024, Wan et al. recently published a series of 526 patients with incomplete cytoreduction (44% CCR2, 56% CCR3). HIPEC with mitomycin (33%) or cisplatin (42%) was performed in 77% of cases.^32^ The 5‐ and 10‐year survival rates of patients after incomplete CRS treated with HIPEC were significantly higher than those without HIPEC (5y—OS: 58% vs. 48%, 10y—OS: 37% vs. 16%, *p* = 0.032). Multivariate analysis showed that CRS without HIPEC and high pathological grade were independent risk factors for poor prognosis (*p* = 0.007, *p* = 0.0001). Postoperative outcomes or morbidity of HIPEC were not explored in this analysis.

Therefore, HIPEC associated with incomplete CRS could enhance oncological outcomes in patients with low‐grade PMP. For others, further studies are needed.

### 3.3 PIPAC

Concerning peritoneal metastasis, pressurized intraperitoneal aerosol chemotherapy (PIPAC) offers a new treatment option that could impact PMP treatment in the future.^50^ Reymond et al. provided clear recommendations with regard to operative technique, safety checklist and treatment protocols.^51^ Recent Consensus statement for treatment protocols has been published.^52^, ^53^ Developed drugs regimen are numerous.^54^, ^55^, ^56^ In other malignancies PIPAC has shown interesting data concerning toxicity, feasibility, and safety.^56^, ^57^ PIPAC could be a step to complete CRS + HIPEC surgery in unresectable peritoneal metastases (PM) as reported by Alayami et al. achieving a 14% complete resection rate.^58^ PIPAC could be alternate with systemic chemotherapy thus improving quality of life.^59^

Nonetheless, PIPAC experience in PMP is scarce. Encouraging results about PIPAC motivated the creation of an international registry (NCT03210298). Concerning peritoneal metastasis, PIPAC is therefore a hot topic nonetheless few studies included carcinomatosis secondary to pseudomyxoma. Efforts should be made in this direction.

### 3.4 Intravenous chemotherapy

Concerning patients with unresectable low‐grade PMP, a recent US registry involving a cohort of 639 patients reported no benefit in overall survival for the subgroup of 431 patients who received systemic chemotherapy.^60^ In 2023, Ghelardi et al. reported a series of 15 patients with recurrent or unresectable PMP (2 high‐grade) with confirmed disease progression, all received MMC plus metronomic capecitabine (defined as dose‐dense administration at lower doses than the maximum tolerated dose but at shorter free intervals) and bevacizumab until disease progression.^61^ At a median follow‐up of 26 months, median PFS was 17.9 months, with 1‐year PFS and OS rates of 73% and 87%, respectively. The safety profile was manageable with only 13% severe adverse events.

Patients with unresectable high‐grade mucinous PMP, usually received systemic chemotherapy such as intravenous 5 FU, oxaliplatin, and bevacizumab.^62^, ^63^ Lieu et al. reported the results in 78 patients treated primarily for grade 3 PMP. The response rate was 44%, the rate of second‐line administration 57%, the subsequent complete resection rate 33%, progression‐free survival 7.8 months and overall survival 1.7 years.^64^

The MD Anderson cohort of 59 patients highlighted the impact of bevacizumab on progression‐free survival and overall survival, mainly in high‐grade mucinous carcinoma, confirming experimental data in current practice.^62^, ^65^, ^66^ Well‐differentiated PMP benefited less from chemotherapy and bevacizumab than poorly differentiated PMP.

### 3.5 Bromelain

Mucins are a family of high molecular weight, highly glycosylated proteins that may enhance pathological invasion in various neoplasms.^67^, ^68^ It confers chemoresistance by preventing drug penetration and enhancing immune evasion. Bromelain, that is, extracted from the stems of the pineapple plant, and *N*‐acetylcysteine, as a combination is capable of mucin lysis and inhibiting the proliferation and survival of gastrointestinal cancer cells in vitro and in vivo.^69^, ^70^, ^71^ Based on these findings, bromelain and acetylcysteine (BromAc) have been used in clinical settings.

Valle et al. reported treatment with BromAc administered by drain under radiological guidance in 20 patients diagnosed with inoperable PMP or who refused surgery.^72^ At 24 h, patients were assessed for symptoms, including treatment‐emergent adverse events (AEs), then the drain was aspirated. The most common AEs were C‐reactive protein elevation (85%), leukocytosis (55%), and pyrexia (35%). Serious adverse events occurred in 12.5% of patients, with one case of intestinal fistula. The authors raised concerns about the possibility of postoperative intra‐abdominal sepsis or wound infection due to pre‐existing bowel wall erosion from the tumor. An objective response to treatment was seen in 73.2% of treated sites. There was no adverse effect on quality of life in 14 patients. Interestingly, three patients were treated for hepatic hilar lesions, which are known surgical contraindications to complete CRS.

Skalkos et al. reported the case of a 64‐year‐old man diagnosed with appendiceal PMP complicated with gastric outlet obstruction from a perigastric tumor deposit treated by BromAc.^73^ An 80% reduction in size was achieved with the resolution of symptoms.

Rodríguez‐Ortiz et al. conducted a phase Ib/II study to evaluate the safety and efficacy of intratumoral administration of BromAc according to a standardized protocol.^29^ Nine patients with a previous surgical history of CRS were treated for 11 different recurrent masses. Three of the nine patients received a second treatment. CT scan evaluation at 1 month showed encouraging results with 54.5% partial response and stabilization at 12 months follow‐up. During follow‐up, three patients died due to disease progression. Reports of long‐term treatment up to 4 years with BromAc are increasing.^74^

Future protocols of BromAc associated in the perioperative courses with CRS and HIPEC or EPIC or PIPAC should be studied in the future.^75^ Concerns about the anticoagulant or proteolytic effects of bromelain may limit its use in the perioperative setting. However, no hemorrhagic complications have been reported in clinical trials.^72^ Moreover, in vivo models of colonic anastomosis exposed to BromAc showed no interference with the healing process.^76^

While bromelain is an oral drug approved in Germany for the reduction and prevention of edema after surgery or injury, local intraperitoneal administration has been its only use for peritoneal mucinous deposits since its first reports. Recently, Geisel et al. reported the case of a 58‐year‐old man diagnosed postoperatively after laparoscopic appendectomy with pT4a LAMN complicated by disseminated mucinous implants.^77^ The patient refused the committee's recommendation of CRS + HIPEC and opted for oral bromelain and acetylcysteine treatment. Follow‐up to date has been 48 months, including regular magnetic resonance imaging (MRI) scans, with stable findings. This report may broaden our horizons.

### 3.6 Future perspectives

Anti‐inflammatory therapy has reported encouraging results on mucin secretion by downregulation of cytokines in in vitro and in vivo models.^78^ Perioperative anti‐*H. Pylori* therapy may enhance postopererative outcomes.^79^ Immunotoxin therapy such as MOC31PE, targeting a transmembrane epithelial receptor along with a *Pseudomonas* exotoxin, or RDEA119, a MAPK pathway inhibitor, is still in development.^80^, ^81^ Recently, HIF‐1α inhibitors effectively slowed the progression of PMP in mouse xenotransplantation models.^82^ PD‐1 receptors are expressed in 36% of PMP patients, and PD‐L1 is approximately 16%–18%.^83^ Moreover, 6.3% of 155 PMP were recently reported as MMR deficient, encouraging the use of anti‐immunocheckpoint inhibitor in this indication.^84^ Recently, Flatmark et al. proposed a peptide vaccine targeting mutated GNAS for PMP treatment that is still in development.^85^ To conclude, new therapies are coming and doctors must stay alert.

## 4 DECISION‐MAKING FACTORS

Before deciding to carry out CRS + HIPEC, a number of factors need to be evaluated: the patient, the disease, and prognostic indicators.

### 4.1 The patient

The patient's general condition, comorbidities, and nutritional status are all points to consider before proposing a CRS plus HIPEC, which may turn out to be an unnecessary invasive, and costly surgery if the PMP is spread.

### 4.2 The disease

In addition to looking for very rare extra‐peritoneal metastases, the imaging work‐up assesses peritoneal extension and resectability by analyzing the peri‐hepatic peritoneal spaces and the small intestine.

#### 4.2.1 CT scan

A computed tomography (CT) scan is the optimal choice for routine preoperative evaluation and it should be the preferred diagnostic imaging modality.^86^ A mucinous neoplasm is suspected when there is high attenuation of the peritoneal thickening or masses disrupting the natural anatomy on CT scans. Typically, visceral scalloping, particularly of the liver, suggests mucinous ascites. CT scan is known to underestimate the extent of peritoneal metastases, particularly by failing to detect small lesions or lesions with low lesion contrast to adjacent structures such as the small bowel.^87^, ^88^ The sensitivity of CT in detecting peritoneal disease depends on the size and location of the nodules. The reported sensitivity of detecting lesions drops from 59% to 94% for nodules >5 cm to 19%–28% for lesions <1 cm and only 11%–28% for identifying lesions <0.5 cm. Despite this, contrast‐enhanced thoraco‐abdomino‐pelvic CT is the most commonly used test in the preoperative evaluation and follow‐up of patients with PMP.

A scannographic score proposed in 2015 predicts the degree of resectability of low‐grade PMP (simplified preoperative assessment for appendix tumor—SPAAT).^89^ The presence of scalloping in 4 regions (liver, spleen, pancreas, and portal vein) is worth 1 point, while mesenteric retraction is worth 3 points. A high score in one region does not necessarily mean failure of cytoreduction but reflects the extent of the disease and its overall aggressiveness. A score of <3/7 predicts complete cytoreduction in 97.1% of cases. Another score has recently been proposed in a bi‐centric study by the RENAPE network, based on the measurement of 5 distances in the peri‐hepatic area, which can predict resectability with a sensitivity of 94% and a specificity of 81% (sum of distances <28 mm).^90^ These scores are easy to obtain, reproducible, and useful in anticipating the surgical project.

The CT scan will also look for associated pleural involvement, most commonly as a result of disease extension secondary to a postoperative diaphragmatic wound or congenital pleuroperitoneal communication.^91^ Extra‐abdominal metastases secondary to hematogenous and/or lymphatic dissemination can be occasionally observed, particularly in the lung.^92^ Finally, CT scan can also be used to assess sarcopenia, which is predictive of overall survival but not the risk of serious complications.^93^

#### 4.2.2 MRI

MRI is increasingly emerging as an essential adjunct for preoperative assessment, comprehensive lesion mapping, and monitoring of patients with PMP.^94^, ^95^, ^96^, ^97^ The fluid nature of peritoneal implants, associated with the presence of mucin, makes them easier to detect on T2‐weighted and diffusion sequences. Low et al. showed that MRI correctly categorized preoperative PCI better than CT.^95^ In Menassel et al.'s study, MRI was particularly useful and effective compared to CT in assessing the hepatic hilum and small bowel invasion, both of which are major risk factors for unresectability.^96^, ^97^ Low et al. also showed that MRI was effective in predicting suboptimal cytoreduction in the presence of a mesenteric mass greater than 5 cm or with proximal mesenteric occlusion, as well as in the presence of diffuse small bowel involvement.

Therefore, recommendations published in 2021 indicated that cross‐sectional imaging with MRI could be one of the diagnostic imaging modalities for patients with appendiceal PMP.^86^

#### 4.2.3 Role of laparoscopy in staging of appendiceal PMP

The extent of disease and, more importantly, the ability to achieve complete cytoreduction determine the appropriateness and efficacy of CRS and HIPEC treatment. The limiting factor is often the small volume or miliary involvement of the small bowel, which is currently not detectable by cross‐sectional imaging. The benefit of exploratory laparoscopy in the staging of peritoneal carcinomatosis has been clearly established in patients with peritoneal metastases from colorectal cancer, albeit with an underestimation of PCI in half of patients.^22^ In particular, it allows accurate assessment of small bowel involvement, which is difficult for radiologists.

However, as almost all PMP patients are destined for surgical exploration by laparotomy with at least incomplete cytoreduction (+/‐HIPEC) in case of unresectability, the benefit of exploratory staging laparoscopy in this disease seems less obvious. In addition, gelatinous ascites sometimes complicates exploration and reduces the yield of the procedure.

One of the advantages of laparoscopy is that tumor material can be obtained to determine the grade of PMP. However, the grade is not evenly distributed throughout the abdomen. Therefore, only if the biopsy shows the presence of high‐grade lesions can primary systemic chemotherapy be discussed if the peritoneal disease is too extensive for CRS.

#### 4.2.4 Prognostic factors

A recent analysis of 2637 CRS and HIPEC for PMP of appendiceal origin showed that elevated tumor markers, PCI, gastrectomy, and tumor grade were independent predictive factors for disease‐free survival, and gender, age, elevated tumor markers, PCI and tumor grade influenced overall survival.^98^ Then, the authors developed a nomogram for OS and DFS that took these different variables into account, constituting a valuable tool for decision‐making. These points have also been demonstrated in a previous study.^99^ Multivariate Cox analysis identified distant metastasis, complete cytoreduction score, tumor histology, HIPEC use, and gender as independently predictive of survival. A prognostic index was derived, and four risk groups were categorized; median survival for the four risk groups differed significantly from 240 months to 19.4 months.

Concerning mutation status, the most frequently identified somatic gene mutations in patients with PMP included KRAS (38%–100%), GNAS (17%–100%), and TP53 (5%–23%); however, there were conflicting results of their effect on survival and they are not currently part of the operative decision.^100^ In a study of 18 PMP published in 2015, KRAS and/or GNAS mutations were common genetic features of PMP, but different molecular signatures were observed between low‐grade and high‐grade PMP, suggesting that mutations in TP53 and/or genes involved in the PI3K–AKT pathway may confer malignant properties to PMP.^101^

## 5 CONCLUSION

Complete cytoreductive surgery followed by hyperthermic intraperitoneal chemotherapy remains the gold standard treatment for PMP, leading to prolonged survival, and if not feasible, incomplete CRS with or without HIPEC can be discussed. Alternative therapeutics involving PIPAC, injection of bromelain, immunotoxin therapies and vaccines are in development and should change the treatment alternatives of PMP.

## 6 CONFLICT OF INTEREST STATEMENT

The authors declare no conflict of interest.

## SYNOPSIS

The treatment of pseudomyxoma peritonei is evolving. Complete cytoreductive surgery followed by hyperthermic intraperitoneal chemotherapy is still the gold standard, but alternatives aimed at achieving complete resection or reducing symptoms have emerged.

## Data Availability

Data sharing is not applicable to this article as no data sets were generated or analyzed during the current study.
